# Treatment Outcomes of Hepatitis C-Infected Patients in Specialty Clinic vs. Primary Care Physician Clinic: A Comparative Analysis

**DOI:** 10.1155/2019/8434602

**Published:** 2019-06-04

**Authors:** Taseen Ahmed Syed, Muhammad Hassaan Bashir, Samid Muhammad Farooqui, Allshine Chen, Sixia Chen, Salman Nusrat, Javid Fazili

**Affiliations:** ^1^Department of Internal Medicine, University of Oklahoma Health Sciences Center, 1100 N Lindsay Ave, Oklahoma City, OK 73104, USA; ^2^Department of Gastroenterology, Virginia Commonwealth University, Richmond, VA, USA; ^3^Department of Gastroenterology, SUNY Health Science Center, Brooklyn, New York, USA; ^4^Department of Biostatistics & Epidemiology, University of Oklahoma Health Sciences Center, 801 NE 13th St., Oklahoma City, OK, USA; ^5^Department of Medicine, Neurogastroenterology and Motility Program, University of Oklahoma Health Sciences Center, Oklahoma City, OK, USA; ^6^Department of Medicine, Section of Digestive Diseases and Nutrition, University of Oklahoma Health Sciences Center, Oklahoma City, OK, USA; ^7^Department of Medicine, Section of Digestive Diseases and Nutrition, Veterans Affairs Medical Center, Oklahoma City, OK, USA

## Abstract

**Background:**

Oral direct-acting antivirals (DAAs) provide an exceptional opportunity to treat hepatitis C virus (HCV) infection.

**Goals:**

We compared the treatment outcomes between specialty and primary care physician (PCP) clinics for patients treated with DAAs.

**Methods:**

We performed a retrospective analysis of patients treated for HCV in our PCP clinics and specialty; liver and gastroenterology clinics and gastroenterology clinics. We used the two-sided *t*-test and the chi-square test to compare the means of continuous and categorical variables, respectively.

**Results:**

Data from a total of 377 patients was analyzed (PCP clinic: *n* = 185 and specialty clinic: *n* = 192). There was no significant difference between age, race, and gender. Model for End-Stage Liver Disease (MELD) and Child-Turcotte-Pugh (CTP) scores were comparable at baseline. Greater than 90% of the patients achieved sustained virological response (SVR) with no difference between the groups.

**Conclusions:**

Uncomplicated patients can be treated for hepatitis C by their PCPs with DAAs with similar treatment outcomes to specialty clinics. There should be explicit guidelines on patient eligibility for treatment by PCPs vs. specialists.

## 1. Introduction

Novel oral direct-acting antivirals (DAAs) have completely changed the spectrum of hepatitis C treatment with multiple studies showing sustained virological response (SVR) of over 90% for many genotypes [1–3]. Approximately 3.5 million people in the USA are infected with HCV [4]. Since the introduction of DAAs in the late 2013, almost 350,000 patients in the USA have been treated with these agents. This is a small portion of the HCV-infected population which leaves a huge number of treatment-naïve patients who can benefit from this cost-effective treatment [5, 6]. The outpatient setting has always focused on screening, diagnosis, and referring patients for appropriate treatment through specialists including gastroenterologists and hepatologists [7, 8]. There has always been some amount of uneasiness among primary care physicians (general internal medicine and family medicine) [9] for the management of important aspects of HCV-related healthcare [10–12]. Over the last few years, trends have changed from a merely screening-and-referral strategy to a more self-initiated treatment of HCV infection by primary care physicians (PCPs) themselves [13]. One reason for this shifting paradigm was Project ECHO (Extension for Community Healthcare Outcomes), which was launched in 2003 in Mexico and amplified the capacity to practice the best medicine in underdeveloped and rural areas through telemedicine including video conferencing. The purpose of this was to enhance capabilities of PCPs for complex patient care in underserved communities [14]. A subsequent study published by Arora et al. in *New England Journal of Medicine* in 2011 showed that the quality of hepatitis C treatment received by physicians trained through the program ECHO was comparable to specialist's level care [15]. This ECHO act was approved by the US in the late 2016. A similar study recently published in April 2017 used the project ECHO to the VA population (VA-ECHO) [16]. The results were promising and showed that telemedicine through Project ECHO can increase awareness about hepatitis C treatment initiation especially in Veterans Affairs (VA) hospitals in far rural areas. The ECHO project is cost-effective and stresses the fact that chronic HCV infection burden in the US can be reduced by increasing awareness of hepatitis C treatment in the primary care setting [17–19]. The successful implementation of this project in multiple states has proven the utility of PCPs to treat HCV infection through expanding their educational capacity and increasing their comfortability level [15, 20]. The purpose of our study was to assess outcomes of PCPs treating HCV-infected US veterans with DAAs after the incorporation of educational initiatives and collaborations with specialists.

## 2. Methods

We performed a retrospective chart review to identify patients treated for HCV from January 2014 to May 2017, at our PCP clinics and specialty; liver and gastroenterology clinics. The departments of internal medicine, gastroenterology, hepatology, and pharmacy got together to establish a multidisciplinary approach to deal with the shortage of physicians available to treat hepatitis C patients in the DAA era as many of them who were not considered for treatment in the interferon era were now eligible for treatment with DAAs. We had two educational sessions conducted by hepatologists for PCPs on chronic HCV management with DAAs. Afterwards, the PCPs had full support of the pharmacy department in monitoring and dispensing of these medications.

After this intervention and from May 2016 onwards, it was decided to send noncirrhotics to PCP clinics for treatment whereas the specialty clinic was still treating both cirrhotics and noncirrhotics in addition to patients who had coinfection with hepatitis B virus (HBV) and HCV, were human immunodeficiency virus (HIV) infected, had ribavirin in the treatment regimen, or had prior treatment failure.

### 2.1. Study Population

A total number of 731 HCV-infected patients were treated by 62 primary care and 5 specialty physicians at our Veterans Affairs Medical Center. Clinical outcomes of patients treated in the PCP clinic were compared to patients treated in the specialty clinic. We collected the baseline characteristics (age, gender, race, smoking history, alcohol history, and HCV genotype), baseline HCV RNA level, achievement of SVR, coinfection with hepatitis B or HIV, hepatocellular carcinoma (HCC) development after starting treatment, Model of End Stage Liver Disease (MELD) scores, Child-Turcotte-Pugh (CTP) scores, and other cirrhosis-related pre- and posttreatment laboratory parameters.

### 2.2. Inclusion Criteria

Adults, both men and women, between ages 18-79 years, were included in the study. These patients were infected with HCV and received treatment in their PCP or specialty clinic.

### 2.3. Exclusion Criteria

Patients with loss to follow-up due to transfer of care to another facility, death before SVR, or no show for SVR labs were not included as per protocol.

### 2.4. Statistical Analysis

We used the two-sided *t*-test and the chi-square test to compare the means of continuous and categorical variables, respectively. The analysis was performed with the statistical analysis software (SAS). A *p* value of <0.05 was considered statistically significant. The 95% confidence interval was calculated using a Wald asymptotic 95% confidence interval for difference of two proportions.

### 2.5. Main Outcome Measure

The primary endpoint was SVR (an undetectable HCV viral load 12-week posttreatment), which represents cure. Development of HCC was our secondary endpoint.

## 3. Results

**Figure 1:**
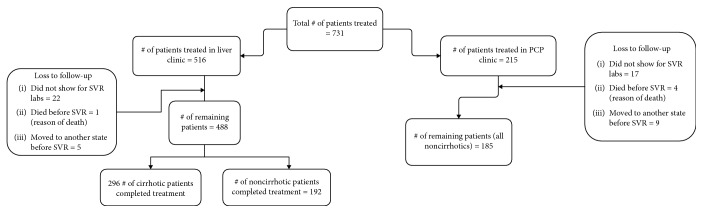
Flow chart.

In total, the data from 673 HCV-infected patients was extracted. 488 patients were treated in the specialty clinic and 185 in the PCP clinic (Figure 1). The rate of SVR was high in the PCP clinic as compared to specialty clinic (93.51 vs. 92.40, *p* = 0.62). Since cirrhotics were being treated in the specialty clinic, they were excluded to prevent any bias, since cirrhosis is an independent risk factor for poor treatment outcomes (decreased SVR rates) [21]. Data from a total of 377 patients was reanalyzed. 192 and 185 noncirrhotics (via biochemical and radiological studies) were treated in the specialty and PCP clinics, respectively. Compliance to medication was noted by regular visits at the time of enrollment, four weeks into treatment (4VR), end of treatment (ETR), and SVR lab withdrawal. Demographic variables are presented in Table 1, and clinical outcomes assessed by the chi-square or *t*-test are presented in Table 2. The groups were comparable in regard to race, gender, MELD, CTP, FIB-4 scores, and posttreatment labs (AST, ALT, hemoglobin, total bilirubin, albumin, and sodium) (Table 2). All patients in both groups completed therapy. The HCV genotype was known for all patients; 112 (58.33%) specialty clinic and 113 (61.08%) PCP clinic patients had genotype 1a, which was the most common genotype. SVR was achieved in more than 90% of the patients in both groups separately, and there was no statistical difference for SVR results between the groups (Table 2). The risk difference of the proportions of patients achieving SVR in both groups (95.8% in the GI clinic and 93.5% in the PCP clinic) was 2.32% with 95% CI of -2.05% to 6.69%. The number of treatment-naïve patients was higher in the PCP clinic as compared to GI clinic (92.97% vs. 77.60%, *p* = <0.01) (Table 3). The top three drugs used in the PCP clinic were Harvoni, Epclusa, and Zepatier whereas in the GI clinic, Harvoni, sofosbuvir/ribavirin, and Viekira/ribavirin were more often used. 97.56% and 93.06% of patients achieving SVR in the GI and PCP clinics, respectively, received Harvoni as sole treatment (Table 4). Most patients achieving SVR were genotype 1a in both groups (96.4% in the GI clinic vs. 92.92% in the PCP clinic) (Table 5). MELD, CTP, and FIB-4 scores were comparable at SVR. No major complications such as death were reported. Only two patients developed HCC, one from each group. There was no difference in markers of disease severity posttherapy, assessed by MELD, CTP, and FIB-4 scores (Table 2). As far as high-risk behavior was concerned, history of smoking, alcohol intake, and IVDU was more common in patients treated in the GI clinic (*p* < 0.01).

## 4. Discussion

HCV infection has always been a prevalent but less diagnosed viral infection. The reason is the knowledge gap and lack of educational expertise outside of the specialist practice [7, 22]. However, treatment of hepatitis C by a PCP results in less loss to follow-up due to familiarity with patient social dynamics prior to HCV treatment initiation. Treating HCV infection themselves increases their awareness of liver diseases and is a source of intellectual satisfaction that can prevent burn out as well [23]. Contrary to this, PCPs have to face certain drawbacks. PCPs have to treat patients in entirety and thus put a knowledge burden on them. HCV treatment is often restricted by payers to patients with advanced fibrosis forcing PCPs to perform additional tests like a fibroscan that requires another complex level of understanding [24, 25]. Time restraints, knowledge gaps, easy access to the specialty referral, apprehension of medicolegal litigation, lack of HCV treatment in PCP quality measures, and provider restrictions on the prescription of independent HCV treatment are some of the reasons why PCPs are not comfortable treating hepatitis C [23, 26, 27]. Definitely, voluntary participation by PCPs is still the key factor here.

There have been no guidelines on when HCV-infected patients should be referred to the PCP clinic. In our institution, we established our own referral criteria that focused on complex patients being treated in the specialty clinic. The updated guidelines are written by specialists for specialists and are not intended for the PCP audience. When it comes to referral to the specialty clinic, there are no clear indications when PCP should refer to specialists rather than treating themselves [28]. Our study found that HCV-infected patients with mild disease as assessed by MELD score, CTP score, and FIB-4 score can be treated successfully by PCPs with comparable SVR to the specialty clinics. As far as referral is concerned, guidelines based on firm evidence and extensive research with clear-cut indications on when to request a specialist referral should be outlined.

Finally, the elimination of hepatitis C is a public health goal and to achieve, it will require a multidisciplinary approach with hepatitis C treatment awareness and access to multiple health care workers in health care settings including PCP clinics, pharmacist clinics, methadone clinics, and prisons. In our clinics, pharmacists played a role of a clinical and specialty pharmacist and as such, assisted physicians in prior medication authorization, prescription fulfillment, and patient telephonic counseling. On the other hand, a health system specialty pharmacist can be directly involved in hepatitis C treatment initiation further reducing the burden of hepatitis C management that has shown promising results [29, 30]. Since different DAAs are selected based on their pharmacodynamics, adverse effects, efficacy, cost implications, and insurance approval, the role of pharmacists can be promising to enhance treatment access. By the participation of PCPs and pharmacists, the WHO goals for viral hepatitis can be achieved.

### 4.1. Study Limitations

There are several limitations to this study. This study took place in different clinics (PCP vs. GI) but in one hospital setting. Thus, these findings may not be generalizable to all settings. We did not directly study the parameters for nonadherence rather considered SVR to be a marker of adherence to the treatment regimen. As compared to PCP clinics, the specialty clinics treated more complicated patients with past high-risk lifestyle habits including smoking, alcohol intake, and intravenous drug use. These lifestyle differences might be a potential cause of confounding.

## 5. Conclusions

HCV patients with mild disease severity who were treated in the PCP clinics achieved SVR at rates comparable to HCV patients treated in the specialty clinics. This high SVR was achieved through a multidisciplinary approach including PCPs. Uncomplicated patients can be treated for hepatitis C by their PCPs with safe and cost-effective DAAs and thereby relieve the heavy burden on the specialty clinics especially in Veterans Affairs (VA) Health which is considered to be the world's largest hepatitis C care provider. However, further educational initiatives and explicit practice guidelines for PCPs are needed.

## Figures and Tables

**Table 1:** Demographic values by clinic with the chi-square test or the *t*-test.

Demographic variables	GI clinic (*n* = 192)	PCP clinic (*n* = 185)	*p* value
Age, average (range)	60.71 (29-78)	61.54 (30-87)	0.27
Male sex, *n* (%)	180 (94.74%)	182 (98.38%)	0.05
Race, *n* (%)			
(a) Caucasian	130 (73.00%)	114 (64.41%)	0.22
(b) African American	42 (23.60%)	55 (31.07%)	
(c) Other^∗^	6 (3.37%)	8 (4.52%)	
(d) Unknown	14 (7.29%)	8 (4.52%)	
Cirrhosis, *n* (%)	0 (0.0%)	0 (0.0%)	
Comorbidities			
(a) CHF, *n* (%)	5 (2.63%)	12 (6.52%)	0.07
(b) HTH, *n* (%)	123 (64.40%)	123 (66.85%)	0.62
History of the previous alcohol use, *n* (%)	178 (94.68%)	109 (62.29%)	<0.01
History of the previous smoking, *n* (%)	133 (70.74%)	48 (29.09%)	<0.01
History of the previous IVDU, *n* (%)	115 (61.17%)	46 (27.38%)	<0.01

^∗^Other: Native American and Asian. Abbreviations: GI: gastroenterology; PCP: primary care physician; CHF: congestive heart failure; HTN: hypertension; IVDU: intravenous drug use.

**Table 2:** Outcomes by clinic with the chi-square test or *t*-test.

Covariate	GI clinic (*n* = 192) before SVR	PCP clinic (*n* = 185) before SVR	Parametric *p* value^∗^	GI clinic (*n* = 192) at SVR	PCP clinic (*n* = 185) at SVR	Parametric *p* value^∗^
Patients achieving SVR, *n* (%)				184 (95.83%)	173 (93.51%)	0.32
MELD score, mean (range)	7.32 (6.96-7.68)	7.87 (7.39-8.34)	0.27	8.50 (6.00-26.00)	8.41 (6.00-24.00)	0.93
CTP score, mean (range)	5.08 (5.03-5.13)	5.14 (5.09-5.20)	0.08	5.39 (5.00-11.00)	5.22 (5.00-8.00)	0.44
Fib-4 score, mean (range)	1.83 (1.71-1.95)	1.96 (1.84-2.07)	0.14	1.80 (0.53-13.14)	1.63 (0.69-5.18)	0.19
ALT, mean (range)	56.41 (50.04-62.79)	49.55 (44.51-54.60)	0.10	21.87 (6.00-60.00)	22.99 (7.00-173.00)	0.44
AST, mean (range)	45.97 (42.04-49.91)	45.51 (42.16-48.87)	0.86	25.87 (14.00-83.00)	27.29 (10.00-89.00)	0.25
Hemoglobin, mean (range)	14.86 (14.66-15.06)	14.84 (14.60-1t5.08)	0.91	14.71 (8.30-17.80)	14.65 (10.90-18.70)	0.75
T. bilirubin, mean (range)	0.80 (0.75-0.84)	0.75 (0.70-0.79)	0.10	0.71 (0.20-4.60)	0.65 (0.10-2.30)	0.18
Albumin, mean (range)	4.03 (3.98-4.07)	3.91 (3.85-3.97)	<0.01	3.99 (1.21-5.00)	4.00 (0.30-4.90)	0.98
Na, mean (range)	138.1 (137.8-138.5)	136.9 (136.5-137.4)	<0.01	137.7 (128.0-143.0)	137.2 (127.0-143.0)	0.08
Reported HCC posttherapy, *n* (%)				1 (0.52%)	1 (0.54%)	1.00

Abbreviations: GI: gastroenterology; PCP: primary care physician; SVR: sustained virological response; MELD: Model for End-Stage Liver Disease; CTP: Child-Turcotte-Pugh; AST: aspartate aminotransferase; ALT: alanine aminotransferase; HCC: hepatocellular carcinoma.

**Table 3:** Prior hepatitis C treatment regimens for patients treated in GI vs. PCP clinic.

Prior treatment	GI clinic (noncirrhotic) Group-1 (*n* = 192)	PCP clinic (noncirrhotic) Group-2 (*n* = 185)	*p* value
Treatment naïve, *n* (%)	149 (77.60)	172 (92.97)	<0.01
Previous treatment, *n* (%)	43 (22.40)	13 (7.03)	
Interferon+ribavirin	33 (17.19)	11 (5.95)	<0.01
PEG/ribavirin/boceprevir	5 (2.60)	1 (0.54)	
Harvoni & ribavirin	4 (2.08)	0	
Viekira Pak+ribavirin	0	1 (0.54)	
Interferon	1 (0.52)	0	

Abbreviations: GI: gastroenterology; PCP: primary care physician; PEG: pegylated interferon alpha.

**Table 4:** SVR outcomes based on the prescribed anti-HCV medication.

Current treatment	GI clinic (noncirrhotic) Group-1 (*n* = 192)	PCP clinic (noncirrhotic) Group-2 (*n* = 185)	*p* value	*n* (%) of patients achieving SVR in GI clinic for the drug	*n* (%) of patients achieving SVR in PCP clinic for the drug
Harvoni	123 (64.06)	144 (77.84)	<0.01	120 (97.56)	134 (93.06)
Sofosbuvir+ribavirin	33 (17.19)	0		32 (96.97)	0
Epclusa	0	25 (13.51)		0	24 (96.00)
Viekira+ribavirin	17 (8.85)	0		15 (88.24)	0
Zepatier	0	15 (8.11)		0	14 (93.33)
Harvoni+ribavirin	9 (4.69)	1 (0.54)		7 (77.78)	1 (100)
Sofosbuvir+Harvoni	3 (1.56)	0		3 (100)	0
Sofosbuvir+simeprevir	1 (0.52)	0		1 (100)	0
Daklinza+sofosbuvir	1 (0.52)	0		1 (100)	0
Sofosbuvir+ribavirin+IFN	1 (0.52)	0		1 (100)	0
Ledipasvir/sofosbuvir	1 (0.52)	0		1 (100)	0
Viekira	1 (0.52)	0		1 (100)	0
Technivie/ribavirin	1 (0.52)	0		1 (100)	0
Harvoni/Viekira/Zepatier	1 (0.52)	0		1 (100)	0

Abbreviations: GI: gastroenterology; PCP: primary care physician; SVR: sustained virological response; IFN: interferon.

**Table 5:** SVR outcomes based on the hepatitis C genotype.

Hepatitis C genotype	GI clinic (noncirrhotic) Group-1 (*n* = 192)	PCP clinic (noncirrhotic) Group-2 (*n* = 185)	*p* value	*n* (%) of patients achieving SVR in GI clinic for the genotype	*n* (%) of patients achieving SVR in PCP clinic for the genotype
1a, *n* (%)	112 (58.33)	113 (61.08)	<0.01	108 (96.43)	105 (92.92)
1b, *n* (%)	32 (16.67)	50 (27.03)		31 (96.88)	47 (94.00)
2b, *n* (%)	25 (13.02)	12 (6.49)		25 (100)	11 (91.67)
2, *n* (%)	8 (4.17)	1 (0.54)		8 (100)	1 (100)
4, *n* (%)	4 (2.08)	0		4 (100)	0
3a, *n* (%)	3 (1.56)	3 (1.62)		2 (66.67)	3 (100)
1, *n* (%)	3 (1.56)	0		2 (66.67)	0
2a/2c, *n* (%)	2 (1.04)	2 (1.08)		2 (100)	2 (100)
3, *n* (%)	2 (1.04)	0		1 (50.00)	0
1a/1b, *n* (%)	1 (0.52)	2 (1.08)		1 (100)	2 (100)
2b & 3, *n* (%)	0	1 (0.54)		0	1 (100)
4a/4c/4d, *n* (%)	0	1 (0.54)		0	1 (100)

Abbreviations: GI: gastroenterology; PCP: primary care physician; SVR: sustained virological response.

## Data Availability

Not applicable. We do not have publicly achieved datasets.
